# Digital breast tomosynthesis (DBT) plus synthesised two-dimensional mammography (s2D) in breast cancer screening is associated with higher cancer detection and lower recalls compared to digital mammography (DM) alone: results of a systematic review and meta-analysis

**DOI:** 10.1007/s00330-021-08308-8

**Published:** 2021-10-25

**Authors:** Sylvia H. Heywang-Köbrunner, Alexander Jänsch, Astrid Hacker, Sina Weinand, Tobias Vogelmann

**Affiliations:** 1 National Reference Centre Mammography Munich, Sonnenstraße 29, 80331 Munich, Germany; 2 FFB gGmbH, Munich, Germany; 3 LinkCare GmbH, Stuttgart, Germany

**Keywords:** Systematic review, Meta-analysis, Mammography, Breast neoplasms, Early detection of cancer

## Abstract

**Objectives:**

Digital breast tomosynthesis (DBT) plus digital mammography (DM) in screening is problematic due to increased radiation by the double exposure. Synthesised two-dimensional mammography (s2D) calculated from DBT datasets at no additional dose appears a sensible alternative compared to adding DM. This systematic review and meta-analysis focuses on screening performance outcomes in women screened with DBT plus s2D compared to DM alone.

**Methods:**

PubMed was searched from January 1, 2010, to September 2, 2020. Studies comparing DBT plus s2D to DM alone in breast cancer screening were included. Pooled risk ratios (RR) were estimated for cancer detection rates (CDR), recall rates, interval cancer rates (ICR), biopsy rates, and positive predictive values for recalls (PPV-1), for biopsies recommended (PPV-2), and for biopsies performed (PPV-3). Sensitivity analyses were performed using the leave-one-out approach. Risk of bias (RoB) was assessed using the Quality Assessment of Diagnostic Accuracy Studies (QUADAS)-2 tool.

**Results:**

Twelve papers covering 414,281 women were included from 766 records identified. CDR is increased ([RR, 95% CI] 1.35, 1.20–1.52), recall rates are decreased (0.79, 0.64–0.98), and PPV-1 is increased (1.69, 1.45–1.96) when using DBT plus s2D compared to DM alone. ICR and biopsy rates did not differ, but PPV-2 respectively PPV-3 increased with DBT plus s2D (1.57, 1.08–2.28 respectively 1.36, 1.17–1.58). Overall RoB of studies was assessed to be low.

**Conclusion:**

Results show improved diagnostic outcomes with DBT plus s2D compared to DM alone and underline the value of DBT in combination with s2D in breast cancer screening.

**Key Points:**

• *DBT plus s2D is associated with higher CDR, lower recall rates, and a higher PPV-1 compared to DM alone in breast cancer screening.*

• *No differences in biopsy rates were found between screening modalities, but PPV-2 and PPV-3 were higher in women screened with DBT plus s2D compared to DM alone.*

• *We identified inconsistent results of ICR in two studies comparing DBT plus s2D to DM alone—resulting in no differences when pooling ICR in meta-analysis.*

**Supplementary Information:**

The online version contains supplementary material available at 10.1007/s00330-021-08308-8.

## Introduction

Female breast cancer is the most common cancer worldwide and surpassed lung cancer in 2020 [1]. Population-based breast cancer screening programmes have been implemented to reduce mortality by early cancer detection. DM represents the current standard in most screening programmes. In the last decade, technological advances in image acquisition resulted in the development of DBT. DBT allows for the calculation of (mostly) 1 mm mammographic slices of the 3D volume imaged in a desired projection. This avoids superimposition, enhances cancer detection, and improves the sensitivity compared to DM. Based on the technology available at that time, initial studies compared the combination of DBT plus DM to DM alone. While the combination proved to be more sensitive [2–4] and more specific [4] than DM alone, it is associated with a ~ 2–2.4-fold dosage compared to DM alone [5–8]. The addition of DM to DBT compared to DM alone may allow for easier assessment of breast symmetry and facilitate the comparison with prior mammograms. Both may yield diagnostically important additional information. s2D from DBT images has been developed as a substitute for the additional DM [6, 9]. Synthetic mammographic images are calculated from the DBT dataset, requiring no double exposure. Thus, the combination of DBT plus s2D promised to maintain the diagnostic advantages of the new method at a radiation dose which is comparable to or only slightly higher than that of DM.

While the diagnostic superiority of DBT plus DM compared to DM alone has already been shown in several meta-analyses [2–4, 10–12], we identified two meta-analyses [13, 14] evaluating DBT plus s2D compared to DM alone. Giampietro et al. [14] estimated an overall higher CDR when using DBT plus s2D compared to DM, whereas no statistically significant differences were observed for recalls. According to Alabousi et al. [13], DBT plus s2D is associated with higher CDR, lower recall rates, and higher PPV-1 compared to DM alone [13]. The differing results of these meta-analyses may be explained by methodological differences. Since differences in recall rates are inconsistent between studies [14], sensitivity analyses are needed to investigate the effect of each study included. Hitherto, no meta-analysis has considered the outcomes of biopsy rate, PPV-2, and PPV-3.

We aimed to perform a systematic review and meta-analysis of CDR and recall rates, focusing on available information on biopsy rates, PPV-1, -2, and -3, and of ICR, limited to studies comparing DBT plus s2D to DM alone. Furthermore, we planned to systematically perform sensitivity analyses to address heterogeneity among studies.

## Materials and methods

### Literature search

A systematic literature search was performed to identify relevant studies published on PubMed between January 1, 2010, and September 2, 2020. Search terms are available in ESM, S1. The search was limited to studies published in English language and with available abstracts. This study is reported in accordance to the Preferred Reporting Items for Systematic reviews and Meta-Analysis (PRISMA) guidelines [15, 16]. Our protocol was registered on INPLASY under registration number INPLASY202140073.

### Eligibility criteria

Prospective and retrospective studies with a comparative design reporting original data were evaluated eligible if, first, asymptomatic women with an average risk of breast cancer presenting for screening were considered, including women with symptoms at a regular screening. No restriction regarding age, gender, or country was set (***P****atients*)*.* Second, studies were included if DBT plus s2D (***I****ndex test*) was compared to DM alone (***C****omparator*)*.* Third, at least data of CDR, recall rates, ICR, biopsy rates, or PPV 1–3 (***O****utcomes*) had to be reported. Studies without human subjects and studies evaluating solely women with symptoms, findings suspicious of breast cancer, or screened for diagnostic work-up only were excluded.

### Study selection and data extraction

Records identified through database search were first screened for eligibility based on information provided in title and abstract and second, using the full texts of articles. In case of any disagreement among reviewers, a third reviewer assessed the record and consensus was reached by discussion. Data of studies assessed to be eligible after full-text screening were extracted into a pretested spreadsheet by two independent reviewers. Reviewers were not blinded to the authors and institutions of studies undergoing review. The data extraction spreadsheet was designed according to the checklist of the *data extraction for complex meta-analysis* (*DECiMAL*) guide [17] (ESM, S1 for detailed information).

### Quality assessment

The RoB and applicability were evaluated by two reviewers independently using QUADAS-2 [18]. In case of any disagreement, a third reviewer was asked for assessment and consensus was reached by discussion. Studies were assessed for RoB regarding the dimensions (I) patient selection, (II) index test, (III) reference standard, and (IV) flow and timing. Applicability was evaluated by dimensions I to III.

### Statistical analysis

Since all outcomes were dichotomous, RR with a 95% CI were used as summary statistics (inverse variance) to express the outcome in women screened with DBT plus s2D in relation to women screened with DM alone. *p* values less than 0.05 were defined as an indicator of statistical significance. Heterogeneity among the studies for each outcome was ascertained visually by forest plots and statistically using the Higgins *I*^2^ for quantification inconsistency. Random effects models (REM) were used to pool an effect size when *I*^2^ > 50%—indicating a moderate to high probability of heterogeneity, otherwise (*I*^2^ ≤ 50%) fixed effects models (FEM) were used. Studies reporting outcomes based on the same population were included once, whereas the study with the larger sample size was included. Analysis was conducted using Microsoft Excel and Review Manager 5.4. To address heterogeneity among studies [19] and to test the robustness of results, sensitivity analyses were done using the leave-one-out approach if at least three studies were included in analysis. Each study was excluded once from meta-analysis and results were recorded to verify whether findings are depending on any study. The results are presented in summary tables in ESM, S2.

## Results

### Study selection

**Fig. 1:**
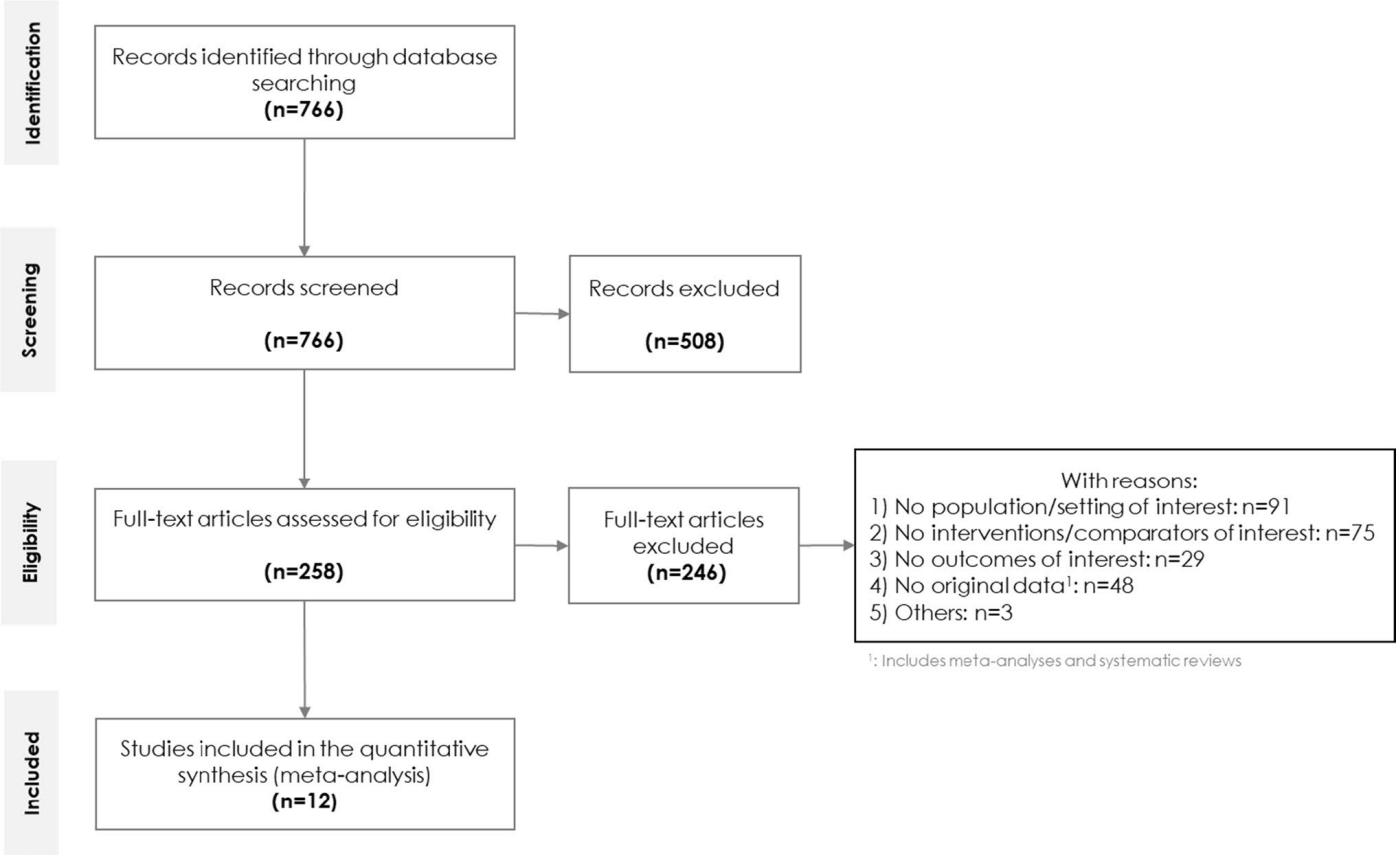
PRISMA flowchart of studies describing the process of selecting studies included in meta-analysis

A total of 766 records were identified in PubMed. Five hundred eight records were excluded after title and abstract screening and 258 records were assessed in full text for eligibility. A total of 246 studies were excluded after full-text screening for eligibility. Twelve studies [5, 20–30], representing the results of 10 populations, were included in at least one meta-analysis of CDR [5, 20–28], recall rate [20, 21, 23–27, 29], ICR [21, 30], biopsy rate [20, 23–25, 27], PPV-1 [20, 21, 23–27, 29], PPV-2 [20, 23], or PPV-3 [20, 23–25, 27]. The selection process is shown in Fig. 1.

### Study characteristics

Table 1 summarises characteristics of studies comparing DBT plus s2D versus DM alone. Twelve studies [5, 20–30] represent results of 10 unique study populations with 414,281 women. Two studies were conducted in the USA (79,209 women) [20, 23], one in Australia (10,146 women) [26], and 9 in Europe, representing results of 7 populations (324,926 women) [5, 21, 22, 24, 25, 27–30]. Two of the European studies reported outcomes from Trento, Italy (Bernardi et al. 2020 [21] and Bernardi et al. 2016 (STORM-2) [5]). Women who were previously enrolled in STORM/STORM-2 were excluded from analysis in Bernardi et al. (2020).

**Table 1:** Summary of study characteristics of included studies comparing DBT plus s2D versus DM alone

						**Inclusion criteria**				**Number of analysis units**		
**Study**	**Year**	**Country**	**Study design**		**Reading**	**Period**		**Age**^**£**^	**Analysis unit**	**DBT + s2D**	**DM**	**Total**
**Studies from the United States (US)**										***25,698***	***53,511***	***79,209***
Aujero et al. [20]	2017	USA	R	Unpaired	Single	10, 2011–06, 2016 (DM); 08, 2015–06, 2016 (DBT plus s2D)		NA	Women^«^	16,173	32,076	48,249
Freer et al. [23]	2017	USA	R	Unpaired	NA	10, 2013–12, 2015 (DM); 01, 2015–12, 2015 (DBT plus s2D)		NA	Women	9,525	21,435	30,960
**Studies from Europe (EU)**										***182,025***^***≠***^	***192,953***^***≠***^	***324,932***^***≠***^
Bernardi et al. [21]	2020	Italy	P	Unpaired	Double	01, 2013–10, 2014 (DM); 10, 2014–10, 2016 (DBT plus s2D)		50	Women^«^	46,343	37,436	83,779
Caumo et al., A (Verona-SC) [22]	2018	Italy	P	Unpaired	Double	04, 2013–03, 2015 (DM); 04, 2015–03, 2017 (DBT plus s2D)		50	Women	34,071	29,360	63,431
Caumo et al., B (Verona-SC) [29]	2018	Italy	P	Unpaired	Double	04, 2014–03, 2015 (DM); 04, 2015–03, 2016 (DBT plus s2D)		50	Women	16,666	14,423	31,089
Bernardi et al. (STORM-2) [5]	2016	Italy	P	Paired	Double	05, 2013–05, 2015		49	Screens^ϕ^	9,677	9,677	9,677
Romero Martin et al. [27]	2018	Spain	P	Paired	Single^≈^	01, 2015–12, 2016		50	Screens^¤^	16,068	16,068	16,068
Hofvind et al. (OVVV) [24]	2018	Norway	P	Unpaired	Double	02, 2014–01, 2016		50	Women	37,185	61,742	98,927
Hovda et al. (OVVV) [30]	2020	Norway	P	Unpaired	Double	02, 2014–12, 2015 (Round 1); 02, 2016–12, 2017 (Round 2)		50	Women	34,641	57,763	92,404
Hofvind et al. (To-Be) [25]	2019	Norway	RCT	Unpaired	Double	01, 2016–12, 2017		50	Women	14,380	14,369	28,749
Skaane et al. (OTST) [28]	2019	Norway	P	Paired	Single	11, 2010–12, 2012		50	Women	24,301	24,301	24,301
**Studies from Australia (AUS)**										***5,018***	***5,166***	***10,184***
Houssami et al. [26]	2019	Australia	P	Unpaired	Double	08, 2017 – 11, 2018		40	Screens^¦^	5,018	5,166	10,184
							**Total number of analysis units**			***212,741***	***251,630***	***414,325***
							**Total number of women**			***212,710***	***251,611***	***414,281***

The bold numbers represent the subtotals (number of analysis units) for studies from the United States (US), Europe (EU), and Australia (AUS)

*STORM-2*, screening with tomosynthesis or standard mammography-2; *Verona-SC*, Verona screening; *OVVV*, Oslo, Vestfold, and Vestre Viken; *OTST*, Oslo Tomosynthesis Screening Trial; *To-Be*, tomosynthesis trial in Bergen; *R*, retrospective; *P*, prospective

^£^Minimum age of included women

^≠^Number of analysis units from Caumo et al. B (2018) and Hovda et al. (2020) were not included

^≈^Single-reading of DBT plus s2D and double-reading of DM

^«^Number of women is equal to the number of screens

^ϕ^9,672 women, since 5 women had bilateral breast cance

^¤^16,067 women, since one women had bilateral cancer

^¦^10,146 women (DBT plus s2D: 4,993; DM: 5,153), since 38 (DBT plus s2D: 25; DM: 13) women were screened annually had two screening episodes each during the trial

### Quality assessment

**Fig. 2:**
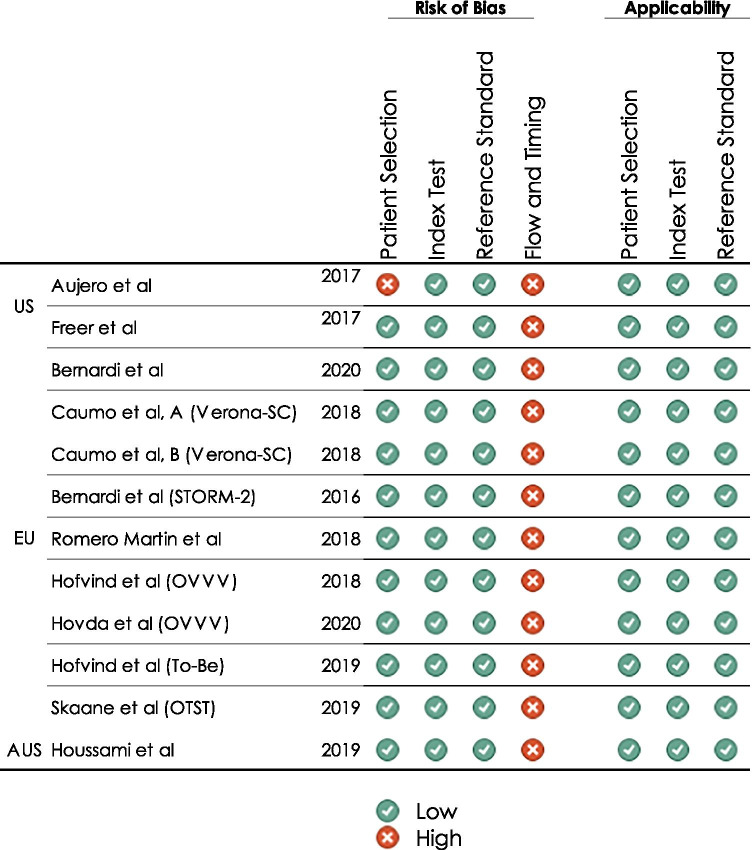
Quality assessment of included studies using QUADAS-2

Figure 2 shows the RoB and applicability assessment. All studies were evaluated having a high RoB in ‘flow of timing’, as not every woman received the same reference test after screening. Low RoB would require that all women, including women with inconspicuous findings in screening, subsequently undergo histopathological assessment for verification. Since this is ethically not acceptable, studies assessed with a high RoB in the domain ‘flow and timing’ **only** were assessed with an overall low RoB (ESM, S3).

### Synthesis of results

#### CDR, recall rate, and PPV-1

**Fig. 3:**
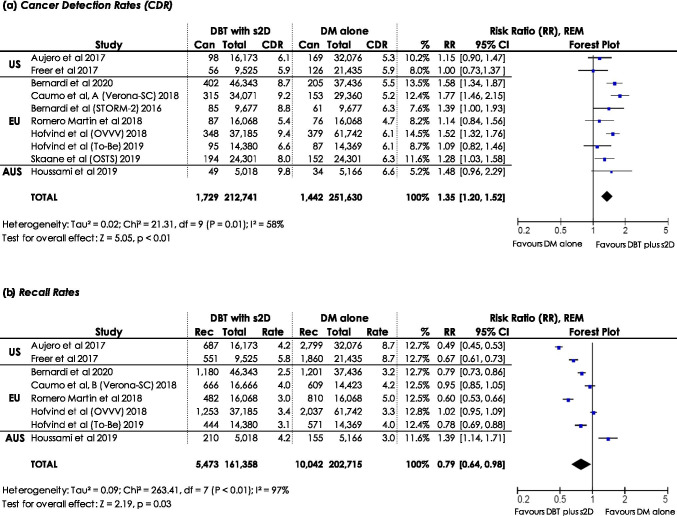
Forest plots for (**a**) cancer detection rates (CDR) and (**b**) recall rates in women screened with DBT plus s2D compared to women screened with DM alone. Squares with horizontal lines represent individual study estimates and 95% confidence interval (CI). Diamond represents the pooled estimate and 95% CI. REM random effects model, Can cancers detected, Rec recalls, % weight

The CDR reports the number of cancers detected among 1,000 women screened/examinations. Ten studies [5, 20–28] were included in the meta-analysis of CDR. Two studies [29, 30] were not included to avoid double counting of women. The CDR was estimated to be significantly higher when using DBT plus s2D compared to DM alone (RR: 1.35, 95% CI: 1.20–1.52, *p* < 0.01, *I*^2^: 58%) using REM (Fig. 3). Sensitivity analyses demonstrated robustness regarding statistical significance (ESM, S2).

The number of women recalled per 100 women/examinations is represented by the recall rate. Eight studies [20, 21, 23–27, 29] were included in the meta-analysis of recall rates (Fig. 3). The study of Bernardi et al. 2016 [5] was excluded since only false-positive recalls were reported. Data of recalls in Skaane (2019) (Oslo Tomosynthesis Screening Trial (OTST)) [28] were not available for the study group DBT plus s2D (Arm D); recalls were reported in total for women screened with DBT plus DM/s2D (Arm C + D). Previous published OTST studies were excluded from analysis, since they did not report recall rates in women screened with DBT plus s2D compared to DM alone [31–33]. Recall rates by REM were significantly lower when using DBT plus s2D compared to DM alone (RR: 0.79, 95% CI: 0.64–0.98, *p*: 0.03, *I*^2^: 97%). Results were not robust with regard to statistical significance if single studies were left out (ESM, S2).

**Fig. 4:**
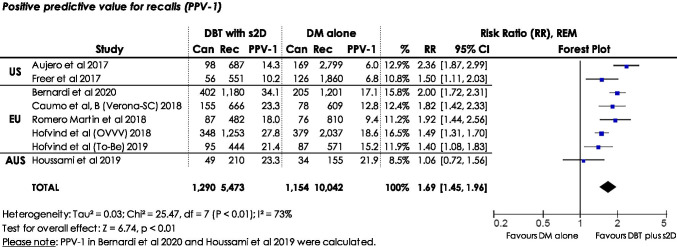
Forest plot for positive predictive value for recalls (PPV-1) in women screened with DBT plus s2D compared to women screened with DM alone. Squares with horizontal lines represent individual study estimates and 95% confidence interval (CI). Diamond represents the pooled estimate and 95% CI. REM random effects model, Can cancers detected, Rec recalls, % weight

The relation of cancers detected by women recalled is represented by the PPV-1. Eight studies [20, 21, 23–27, 29] were included in the meta-analysis of PPV-1 (Fig. 4). Two studies [21, 26] did not separately report the PPV-1; therefore, we calculated PPV-1. There was a statistically significant higher cancer detection when being recalled in screening with DBT plus s2D compared to DM alone (RR: 1.69, 95% CI: 1.45–1.96, *p* < 0.01, *I*^2^: 73%), using REM. Sensitivity analyses demonstrated statistically significant robust results (ESM, S2).

Data of a screening programme in Australia are included in the performed meta-analyses of CDR, recall rate, and PPV-1. Houssami et al. [26] performed sensitivity analyses in which screens of women who reported symptoms at screening were excluded. Symptomatic women are also likely to participate in breast cancer screening programmes; however, the results of the other screening programmes were not stratified for asymptomatic and symptomatic women at screening. Therefore, we included data of all women to enhance comparability between studies, but performed sensitivity analyses, in which the data of asymptomatic women only were included. Results of sensitivity analyses showed no differences of risk ratios for CDR, recall rates, and PPV-1 (ESM, S2).

#### Biopsy rate, PPV-2, and PPV-3

**Fig. 5:**
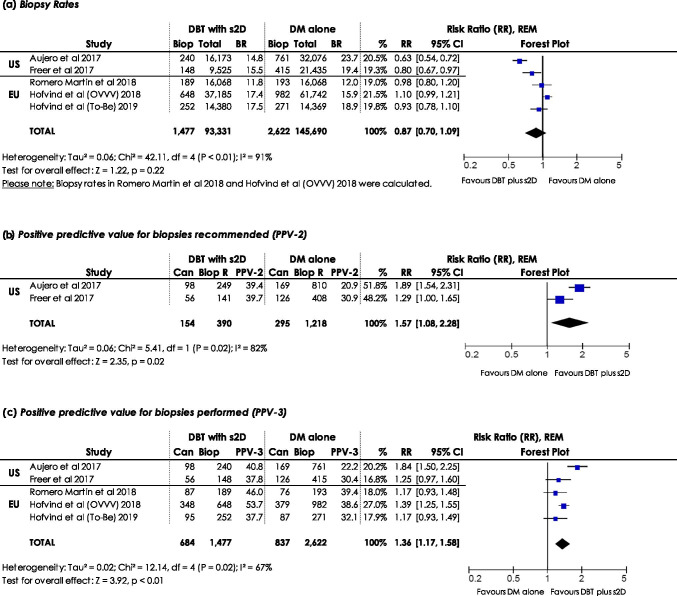
Forest plots for (**a**) biopsy rates, (**b**) positive predictive value for biopsies recommended (PPV-2), and (**c**) positive predictive value for biopsies performed (PPV-3) in women screened with DBT plus s2D compared to women screened with DM alone. Squares with horizontal lines represent individual study estimates and 95% confidence interval (CI). Diamond represents the pooled estimate and 95% CI. REM random effects model, Biop biopsies performed, BR biopsy rate, Biop R biopsies recommended, Can cancers detected, % weight

Biopsy rates indicate how many biopsies were performed per 1,000 women/examinations. Five studies [20, 23–25, 27] were included in the meta-analysis of biopsy rates. In two studies [24, 27], biopsy rates were calculated using the percentage of PPV-3 or CDR. No statistically significant differences in biopsies in women screened with DBT plus s2D compared to DM alone were observed. The RR calculated using REM (RR: 0.87, 95% CI: 0.70–1.09, *p*: 0.22, *I*^2^: 91%) demonstrates a potentially lower number of biopsies when using DBT plus s2D in screening compared to DM alone (Fig. 5). Sensitivity analyses demonstrated robustness of results with regard to statistical significance (ESM, S2).

PPV-2, or PPV-3 respectively, indicates the number of cancers detected among 100 biopsies recommended, or performed, respectively. Since some studies report PPV-2 or PPV-3 only, we analysed both. Five studies were included in the meta-analysis of PPV-3 [20, 23–25, 27]. Cancer detection in women being biopsied after screening with DBT plus s2D is statistically significantly higher (RR: 1.36, 95% CI: 1.17–1.58, *p* < 0.01) compared to women screened with DM alone (Fig. 5), using REM (*I*^2^: 67%). Sensitivity analyses demonstrated robustness of results regarding statistical significance (ESM, S2). PPV-2 (Fig. 5) was reported in two studies [20, 23]. CDR is higher in women with recommended biopsy after screening with DBT plus s2D compared to DM alone using REM (RR: 1.57, 95% CI: 1.08–2.28, *p*: 0.02, *I*^2^: 82%).

#### ICR

**Fig. 6:**
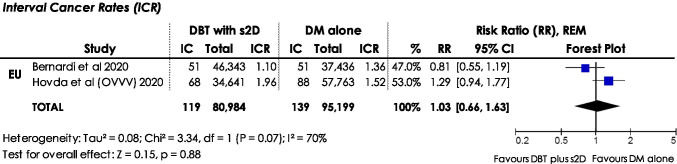
Forest plots for interval cancer rates (ICR) in women screened with DBT plus s2D compared to women screened with DM alone. Squares with horizontal lines represent individual study estimates and 95% confidence interval (CI). Diamond represents the pooled estimate and 95% CI. REM random effects model, IC interval cancers, % weight

The ICR indicates the number of interval cancers per 1,000 women screened/examinations. Two European studies [21, 30] were identified reporting interval cancers in women screened with DBT plus s2D compared to women screened with DM alone (Fig. 6). No statistically significant difference in ICR was observed for both the pooled estimate using REM (RR: 1.03, 95% CI: 0.66–1.63, *p*: 0.88, *I*^2^: 70%) and single ICR reported in the studies.

## Discussion

### Summary of results

Twelve studies comparing DBT plus s2D to DM alone in screening were included in our meta-analyses. We found that screening with DBT plus s2D compared to DM alone is associated with a higher CDR ([RR, 95% CI] 1.35, 1.20–1.52), decreased recalls (0.79, 0.64–0.98), and a higher cancer detection among recalls (1.69, 1.45–1.96). Cancer detection after recommended and performed biopsies was higher with DBT plus s2D compared to DM alone (PPV-2: 1.57, 1.08–2.28; PPV-3: 1.36, 1.17–1.58). We did not identify any differences in biopsy rates and ICR.

### Results interpretation and comparison with literature

Our results regarding CDR, recall rates, and PPV-1 were in line with Alabousi et al. [13]. In comparison to Giampietro et al. [14], we found a statistically significant difference in recalls with fewer recalls for DBT plus s2D than for DM alone. However, the inclusion criteria of the latter study differ. The better results in our and Alabousi’s study may indicate lower recall rates for DBT plus s2D than for DBT plus DM. They may also reflect the learning curve from prior to more recent studies. Statistical significance was lost in our sensitivity analyses of recalls, if, e.g. the US studies Aujero et al. [20] or Freer et al. [23] were excluded. Since screening characteristics like reading procedure or screening intervals differ in the USA, these could be potential factors impacting heterogeneity. In contrast to the other studies, Houssami et al. [26] reported a statistically significantly higher risk of being recalled for one pilot screening trial when using DBT plus s2D compared to DM alone. In this population, women screened with DBT plus s2D were younger, reported symptoms more often, and participated more often in the prevalent screening round compared to women screened with DM alone [26]. Even if recall rates are contrary between studies, the number of cancers detected per 100 recalled women is consistently higher in women screened with DBT plus s2D. DBT plus s2D is associated with higher CDR and concurrently with fewer recalls. Furthermore, higher cancer detection per 100 women with recommended or performed biopsy underlines that DBT plus s2D is more precise in identifying cancers than DM alone. A 9%-point higher sensitivity (83%, 95% CI: 78–87%) for DBT plus s2D compared to DM alone (74%, 95% CI: 65–81%) was also reported by Abdullah et al. [34].

Since high CDR may be related with overdiagnoses, ICR is the more clinically relevant outcome parameter as it reflects potentially important delays in diagnosis and treatment. We identified two European studies [21, 30] reporting ICR comparing DBT plus s2D to DM alone. While Bernardi et al. [21] defined interval cancers as ‘cancers identified over two-year follow-up’ [21], Hovda et al. [30] defined interval cancers as ‘cancers diagnosed 0–24 months after negative screening findings or 6–24 months after false positive baseline screening findings’ [30]. An inconsistent trend of ICR per screening modality and small sample sizes resulted in no statistically significant difference. The same results are shown in a recently published meta-analysis by Houssami et al.[35]. They assessed ICR in women screened with DBT compared to DM. Sensitivity analyses had shown no statistically significant differences in ICR comparing DBT plus s2D to DM alone [35].

Published data on interval cancers following DBT plus s2D are limited and inconsistent. In principle, high CDR and unchanged ICR may be associated with a smaller than expected improvement of mortality reduction and with the risk of increased overdiagnosis. However, the effect of over-detection on mortality reduction and the risk of overdiagnosis can only be estimated after results of follow-up rounds, cancer stages, and biology become available from appropriately designed studies. For improved interpretation of the increased CDR, cancer biology and results for different breast densities may play a role. While Winter et al. [36] reported a lower rate of node positive interval cancers after DBT screening, Bahl et al. [37] reported comparable biology of interval cancer after DBT versus FFDM. Both of these study designs, however, cannot exclude bias. One very recent study, the only study using wide-angle DBT (without s2D), presented reduced ICR after screening with DBT compared to DM [38]. While differences might be associated with the different technologies, possible bias in the control group must also be discussed. Considering the differing results of the limited data on interval cancers, a possible correlation between the amount of recall reduction, additional detection, and effect on ICR might also be worth discussing. Also, most of the included studies were originally not designed and powered to show differences in ICR. Meta-analyses with pooled estimates based on data from underpowered studies with small sample sizes are also likely to be underpowered [39]. Finally, results concerning additional detection and effect on ICR may vary for different ranges of breast densities. To date, these data are not yet available from large studies. Given the fact that the European Commission recommends mammography screening for women aged 45–49 years old [40], DBT could be a more effective alternative, since younger women tend to have more dense breasts and the accuracy of mammography may be poorer. Furthermore, overdiagnoses may be lower in younger women with a longer remaining life-time, as small cancers have a higher risk, or longer time, respectively, for negative development.

### Biases and limitations

This study has several limitations. First, search was carried out in only PubMed and studies that did not have available abstracts and English full text were excluded. Second, in 9 of the ten underlying trials women were not assigned randomly to screening modalities (concerning ~ 385,532 women among a total of 414,281). Also, 4 of the ten underlying trial study groups differ in time periods (concerning ~ 226,419 women among a total of 414,281). Unpaired and non-randomised study designs, for example in which participant characteristics (e.g. breast density, family history, or availability of screening modalities) may differ, lead to potential bias by confounding variables. Since a systematic assessment of potential confounding variables was beyond the scope of our work, a comparison of screening performance of modalities in women with dense breast tissue only seems to be useful in further subgroup analyses. Third, heterogeneity among studies was observed. We used REM and strived to interpret results only considering potential factors impacting heterogeneity. However, our study did not address other influencing or limiting factors. Valuable further data from randomised designs will become available in the near future, for example from a large RCT for which the recruitment of a prospectively acquired study population of 80,000 women [41] was recently completed.

## Conclusion

To our best knowledge, this is the first meta-analysis of biopsy rates, PPV-2, and PPV-3 in women screened with DBT plus s2D compared to DM alone. Statistically significant differences in favour of DBT plus s2D compared to DM alone were found for CDR, recall rates, PPV-1, PPV-2, and PPV-3. Biopsy rates and ICR did not differ between screening modalities. Further research regarding ICR stratified by age and breast density is needed.

## Supplementary Information

Below is the link to the electronic supplementary material.

Supplementary file1 (DOCX 96 KB)

## Abbreviations

CDR: Cancer detection rate
CI: Confidence interval
DBT: Digital breast tomosynthesis
DM: Digital mammography
FEM: Fixed effects model
ICR: Interval cancer rate
INPLASY: International Platform of Registered Systematic Review and Meta-analysis Protocols
OTST: Oslo Tomosynthesis Screening Trial
PPV-1: Positive predictive value for recalls
PPV-2: Positive predictive value for biopsies recommended
PPV-3: Positive predictive value for biopsies performed
PRISMA: Preferred Reporting Items for Systematic reviews and Meta-Analysis
QUADAS: Quality Assessment of Diagnostic Accuracy Studies
REM: Random effects model
RoB: Risk of bias
RR: Risk ratio or relative risk
s2D: Synthesised two-dimensional mammography

## Declarations

### Guarantor

The scientific guarantor of this publication is Prof. Dr. Sylvia H. Heywang-Köbrunner.

### Statistics and biometry

Authors Tobias Vogelmann and Sina Weinand have significant statistical expertise.

### Methodology

• Systematic review

• Meta-analysis
