# Supplementary material for: Digital breast tomosynthesis (DBT) plus synthesised two-dimensional mammography (s2D) in breast cancer screening is associated with higher cancer detection and lower recalls compared to digital mammography (DM) alone: results of a systematic review and meta-analysis
Source: Eur Radiol. 2021 Oct 25;32(4):2301–12. doi: 10.1007/s00330-021-08308-8 (PMC8921114; doi:10.1007/s00330-021-08308-8)
Supplement: Supplementary file 1 — Supplementary file1 (DOCX 96 KB) [file 330_2021_8308_MOESM1_ESM.docx]

**Electronic supplementary material (ESM) 1**

**Search strategy - PubMed**

| #1 | (3d OR 3-d OR “three dimension” OR “3 dimension” OR "Imaging, Three-Dimensional"[Mesh]) |
| --- | --- |
| #2 | (mammography[MeSH] OR "Mammography" OR „Mammographies") |
| #3 | "Tomosynthesis" OR (“selenia dimensions” OR mammomat OR novation OR senoclaire OR “seno claire aspire innovality” OR “aspire f” OR “aspire s” OR “phillips microdose”) |
| #4 | “sensitivity” OR “specificity” OR “accuracy” OR “positive predictive” OR “negative predictive” OR "NPV" OR "PPV" OR “positive test” OR “negative test” OR (“false positive” OR “false negative” OR "area under the curve" or "area under curve" or "receiver operat*") |
| #5 | cancer-detection rate OR cancer detection OR recall rate OR interval cancers |
| #6 | ("breast neoplasms"[MeSH Terms] OR ("breast" AND "neoplasms") OR "breast neoplasms" OR ("breast" AND "cancer") OR "breast cancer") |

= (((#1 AND #2) OR #3) AND (#4 OR #5)) AND #6 AND hasabstract[text] AND English[lang] AND ("2010/01/01"[PDAT] : "3000/12/31"[PDAT])

**Data extracted**

The following data were recorded from each study: (1) study characteristics (including study type and design, geographical location of study sites); (2) information regarding intervention and comparator (including details of screening method, manufacturer and device, screening period, number of women per screening modality, reading procedure); (3) characteristics of study participants (including average age, breast density); (4) outcomes (including CDR (= cancers detected per 1,000 screening examinations respectively women), recall rate (= recalls per 100 screening examinations respectively women), ICR (= interval cancers per 1,000 screening examinations respectively women), biopsy rate (= biopsies per 1,000 screening examinations respectively women), PPV-1 (= cancers detected per 100 recalls), PPV-2 (= cancers detected per 100 biopsies recommended), PPV-3 (= cancers detected per 100 biopsies performed)).

**Electronic supplementary material (ESM) 2**

1. **Results of sensitivity analyses using the leave-one-out approach**

Sensitivity analyses for Cancer Detection Rates using the leave-one-out approach.

|  | **Analysis model** | | **RR** | **95% CI** | **p** | **I²** | **df** |
| --- | --- | --- | --- | --- | --- | --- | --- |
|  | All studies included | REM | 1.35 | [1.20, 1.52] | <0.01 | 58% | 9 |
| **Removed study** | |  |  |  |  |  |  |
| US | Aujero et al 2017 | REM | 1.38 | [1.22, 1.56] | <0.01 | 57% | 8 |
|  | Freer et al 2017 | REM | **1.39** | [1.25, 1.56] | <0.01 | 51% | 8 |
| EU | Bernardi et al 2020 | REM | **1.32** | [1.16, 1.50] | < 0.01 | 58% | 8 |
|  | Caumo et al, A (Verona-SC) 2018 | FEM | 1.36 | [1.26, 1.47] | <0.01 | 46% | 8 |
|  | Bernardi et al (STORM-2) 2016 | REM | 1.35 | [1.19, 1.53] | <0.01 | 62% | 8 |
|  | Romero Martin et al 2018 | REM | 1.37 | [1.21, 1.55] | <0.01 | 59% | 8 |
|  | Hofvind et al (OVVV) 2018 | REM | 1.32 | [1.15, 1.51] | <0.01 | 60% | 8 |
|  | Hofvind et al (To-Be) 2019 | REM | 1.38 | [1.23, 1.56] | <0.01 | 56% | 8 |
|  | Skaane et al (OTST) 2019 | REM | 1.36 | [1.19, 1.55] | < 0.01 | 61% | 8 |
| AUS | Houssami et al 2019 | REM | 1.34 | [1.19, 1.52] | < 0.01 | 62% | 8 |
| REM = random effects model, FEM = fixed effects model, RR = risk ratio, 95% CI = 95% confidence interval, I² = Higgins I² of heterogeneity, df = degrees of freedom, minimum and maximum RR are marked in bold | | | | | | | |

Sensitivity analysis for Recall Rates using the leave-one-out approach.

|  | **Analysis model** | | **RR** | **95% CI** | **p** | **I²** | **df** |
| --- | --- | --- | --- | --- | --- | --- | --- |
|  | All studies included | REM | 0.79 | [0.64, 0.98] | 0.03 | 97% | 7 |
| **Removed study** | |  |  |  |  |  |  |
| US | Aujero et al 2017 | REM | **0.85** | [0.71, 1.01] | **0.06** | 95% | 6 |
|  | Freer et al 2017 | REM | 0.81 | [0.64, 1.03] | **0.09** | 98% | 6 |
| EU | Bernardi et al 2020 | REM | 0.79 | [0.62, 1.02] | **0.07** | 98% | 6 |
|  | Caumo et al, B (Verona-SC) 2018 | REM | 0.77 | [0.61, 0.97] | 0.03 | 98% | 6 |
|  | Romero Martin et al 2018 | REM | 0.82 | [0.66, 1.04] | **0.10** | 98% | 6 |
|  | Hofvind et al (OVVV) 2018 | REM | 0.76 | [0.62, 0.94] | 0.01 | 97% | 6 |
|  | Hofvind et al (To-Be) 2019 | REM | 0.79 | [0.63, 1.00] | **0.05** | 97% | 6 |
| AUS | Houssami et al 2019 | REM | **0.73** | [0.59, 0.91] | < 0.01 | 97% | 6 |
| REM = random effects model, FEM = fixed effects model, RR = risk ratio, 95%-CI = 95% confidence interval, I² = Higgins I² of heterogeneity, df = degrees of freedom, minimum and maximum RR are marked in bold | | | | | | | |

Sensitivity analysis for Positive Predictive Value for Recalls (PPV-1) using the leave-one-out approach.

|  | **Analysis model** | | **RR** | **95% CI** | **p** | **I²** | **df** |  |
| --- | --- | --- | --- | --- | --- | --- | --- | --- |
|  | All studies included | REM | 1.69 | [1.45, 1.96] | < 0.01 | 73% | 7 |  |
| **Removed study** | |  |  |  |  |  |  |  |
| US | Aujero et al 2017 | REM | **1.61** | [1.39, 1.86] | < 0.01 | 65% | 6 |  |
|  | Freer et al 2017 | REM | 1.71 | [1.45, 2.02] | < 0.01 | 76% | 6 |  |
| EU | Bernardi et al 2020 | REM | 1.63 | [1.38, 1.93] | < 0.01 | 70% | 6 |  |
|  | Caumo et al, B (Verona-SC) 2018 | REM | 1.67 | [1.40, 1.98] | < 0.01 | 76% | 6 |  |
|  | Romero Martin et al 2018 | REM | 1.66 | [1.40, 1.96] | < 0.01 | 76% | 6 |  |
|  | Hofvind et al (OVVV) 2018 | REM | 1.73 | [1.46, 2.05] | < 0.01 | 69% | 6 |  |
|  | Hofvind et al (To-Be) 2019 | REM | 1.73 | [1.47, 2.04] | <0.01 | 74% | 6 |  |
| AUS | Houssami et al 2019 | REM | **1.76** | [1.52, 2.04] | <0.01 | 69% | 6 |  |
| REM = random effects model, FEM = fixed effects model, RR = risk ratio, 95% CI = 95% confidence interval, I² = Higgins I² of heterogeneity, df = degrees of freedom, minimum and maximum RR are marked in bold | | | | | | | | |

Sensitivity analysis for Biopsy Rates using the leave-one-out approach.

|  | **Analysis model** | | | **RR** | | **95% CI** | | **p** | | **I²** | | **df** | |
| --- | --- | --- | --- | --- | --- | --- | --- | --- | --- | --- | --- | --- | --- |
|  | All studies included | REM | 0.87 | | [0.70, 1.09] | | 0.22 | | 91% | | 4 | |  |
| **Removed study** | |  |  | |  | |  | |  | |  | |  |
| US | Aujero et al 2017 | REM | **0.96** | | [0.83, 1.10] | | 0.55 | | 68% | | 3 | |  |
|  | Freer et al 2017 | REM | 0.89 | | [0.68, 1.16] | | 0.39 | | 93% | | 3 | |  |
| EU | Romero Martin et al 2018 | REM | 0.85 | | [0.65, 1.11] | | 0.23 | | 93% | | 3 | |  |
|  | Hofvind et al (OVVV) 2018 | REM | **0.82** | | [0.66, 1.01] | | 0.06 | | 83% | | 3 | |  |
|  | Hofvind et al (To-Be) 2019 | REM | 0.86 | | [0.65, 1.14] | | 0.28 | | 93% | | 3 | |  |
| REM = random effects model, FEM = fixed effects model, RR = risk ratio, 95% CI = 95% confidence interval, I² = Higgins I² of heterogeneity, df = degrees of freedom, minimum and maximum RR are marked in bold | | | | | | | | | | | | | |

Sensitivity analysis for Positive Predictive Value for Biopsies performed (PPV-3) using the leave-one-out approach.

|  | **Analysis model** | | | **RR** | | **95% CI** | | **p** | | **I²** | | **df** | |
| --- | --- | --- | --- | --- | --- | --- | --- | --- | --- | --- | --- | --- | --- |
|  | All studies included | REM | 1.36 | | [1.17, 1.58] | | < 0.01 | | 67% | | 4 | |  |
| **Removed study** | |  |  | |  | |  | |  | |  | |  |
| US | Aujero et al 2017 | FEM | **1.31** | | [1.21, 1.43] | | < 0.01 | | 4% | | 3 | |  |
|  | Freer et al 2017 | REM | 1.38 | | [1.15, 1.66] | | < 0.01 | | 74% | | 3 | |  |
| EU | Romero Martin et al 2018 | REM | **1.40** | | [1.18, 1.67] | | < 0.01 | | 70% | | 3 | |  |
|  | Hofvind et al (OVVV) 2018 | REM | 1.34 | | [1.06, 1.69] | | 0.01 | | 75% | | 3 | |  |
|  | Hofvind et al (To-Be) 2019 | REM | **1.40** | | [1.18, 1.67] | | < 0.01 | | 70% | | 3 | |  |
| REM = random effects model, FEM = fixed effects model, RR = risk ratio, 95% CI = 95% confidence interval, I² = Higgins I² of heterogeneity, df = degrees of freedom, minimum and maximum RR are marked in bold | | | | | | | | | | | | | |

1. **Results of the sensitivity analyses excluding women with symptoms at screening reported in Houssami et al 2019**

**Electronic supplementary material (ESM) 3**

Risk of Bias (RoB) assessment in detail of included studies using QUADAS-2.
